# The predictive model for COVID-19 pandemic plastic pollution by using deep learning method

**DOI:** 10.1038/s41598-023-31416-y

**Published:** 2023-03-13

**Authors:** Yaser A. Nanehkaran, Zhu Licai, Mohammad Azarafza, Sona Talaei, Xu Jinxia, Junde Chen, Reza Derakhshani

**Affiliations:** 1 School of Information Engineering, Yancheng Teachers University, Yancheng, 224002 Jiangsu People’s Republic of China; 2 Department of Civil Engineering, University of Tabriz, Tabriz, Iran; 3 Department of Basic Sciences, Maragheh University of Medical Sciences, Maragheh, Iran; 4 School of Informatics, Xiamen University, Xiamen, 361005 Fujian People’s Republic of China; 5 Department of Earth Sciences, Utrecht University, Utrecht, The Netherlands

**Keywords:** Environmental sciences, Public health

## Abstract

Pandemic plastics (e.g., masks, gloves, aprons, and sanitizer bottles) are global consequences of COVID-19 pandemic-infected waste, which has increased significantly throughout the world. These hazardous wastes play an important role in environmental pollution and indirectly spread COVID-19. Predicting the environmental impacts of these wastes can be used to provide situational management, conduct control procedures, and reduce the COVID-19 effects. In this regard, the presented study attempted to provide a deep learning-based predictive model for forecasting the expansion of the pandemic plastic in the megacities of Iran. As a methodology, a database was gathered from February 27, 2020, to October 10, 2021, for COVID-19 spread and personal protective equipment usage in this period. The dataset was trained and validated using training (80%) and testing (20%) datasets by a deep neural network (DNN) procedure to forecast pandemic plastic pollution. Performance of the DNN-based model is controlled by the confusion matrix, receiver operating characteristic (ROC) curve, and justified by the k-nearest neighbours, decision tree, random forests, support vector machines, Gaussian naïve Bayes, logistic regression, and multilayer perceptron methods. According to the comparative modelling results, the DNN-based model was found to predict more accurately than other methods and have a significant predominance over others with a lower errors rate (MSE = 0.024, RMSE = 0.027, MAPE = 0.025). The ROC curve analysis results (overall accuracy) indicate the DNN model (AUC = 0.929) had the highest score among others.

## Introduction

The novel coronavirus disease (2019-nCoV), called by the World Health Organization (WHO) coronavirus disease 2019 (COVID-19), was first reported on December 31, 2019, in Wuhan, China^1^. Due to the increasing number of infected people around the world, the WHO introduced the outbreak of COVID-19 as a pandemic. As of October 10, 2021, more than 240 million people worldwide had been infected by COVID-19, and it had led to the deaths of more than 4.9 million people^2^. The spread of the COVID-19 pandemic affected people's lifestyles in several fields, such as cultural, social, educational, economic, and environmental, which led to more conservative acts. Irreparable damage was caused to the economies of the countries by an increase in the number of patients and the implementation of quarantine^3^. The environmental impact of COVID-19 is also significant. Although the spread of COVID-19 and the forced cessation of the generation of air pollutants, greenhouse gases, industrial wastewater, and wastes, and the cessation of natural resource degradation provided an opportunity for the environment to breathe, the spread of this pandemic produced waste that has unfavorable effects on the environment^4^. Since the outbreak of COVID-19, there has been an unprecedented increase in using single-use plastics (SUPs) such as hand sanitizer bottles, medical test kits, plastic bags, etc. Also, the use of personal protective equipment (PPE), including facemasks, disinfectant wipes, gowns, face shields, and gloves, has been recommended by WHO to decrease the spread of COVID-19 from human to human. Therefore, the management of wastes resulting from SUPs and PPEs is a matter of concern that overshadows global health systems and disrupts the economies of countries. Because of the unusual generation of COVID-19 waste from hospitals and households, a waste emergency has been created on different continents^5^. Since the outbreak of COVID-19, the amount of plastic waste produced worldwide is estimated at 1.6 million tonnes per day, and also almost 3.4 billion facemasks are disposed of daily globally^6^. Table 1 provides information about the number of used facemasks and generated plastic waste during the COVID-19 pandemic. In the meantime, China is estimated to generate nearly 702 million tonnes of plastic waste in the 1st rank in 2020. Iran is located in the 13th rank in the world, generating 62 million tonnes.

**Table 1:** The quantity generated plastic waste during the COVID-19 pandemic in the world.

Continents	Total daily facemask (million)	Plastic waste (ton/day)
Asia	1,876,182,682	954,642
Europe	446,023,935	154,624
Africa	412,815,855	276,466
South america	381,415,704	135,374
North america	245,336,151	75,796
Oceania	21,683,378	8,768

## COVID-19 pandemic wastes

All COVID-19-related waste (SUPs and PPEs) generated by hospitals, health centers, and infected individuals is called COVID-19 pandemic waste. The main of COVID-19 waste includes facemasks, gloves, gowns, face shields used by treatment staff, and types of consumer equipment utilized by COVID-19 patients. It is noteworthy that COVID-19 wastes aren't limited to hospitals and healthcare facilities, but wastes generated by asymptomatic patients or people who have recovered at home are also considered COVID-19 waste. COVID-19-related waste can be divided into two groups, according to the WHO and the US Centres for Disease Control and Prevention (CDC). One is waste generated within healthcare facilities and hospitals, which must be collected with extra care and disposed of as medical waste. Another is waste generated from preventive measures such as facemasks, plastic gloves, etc., which can be considered harmless waste^7^. Solid waste produced via households has gained little notice from the scientific community, while it can facilitate the COVID-19 spread by the fomite transmission route. SARS-CoV-2 can survive on various substrates from a few hours to a few days; for example, it is stable on plastics and stainless steel for 2–3 days^8,9^ Kampf. Therefore, solid waste generated from contaminated PPEs (e.g., gloves and facemasks) is hazardous to health and the environment^10,11^. A patient with COVID-19 can produce about 3.4 kg/day of healthcare waste^12^. The volume of biomedical waste in the form of waste plastics has increased during the COVID-19 pandemic. The facemasks and gloves are mainly composed of non-biodegradable polymeric substances that can degrade as macro-, meso-, and microplastics once discarded into the environment^13,14^. There is an increasing concern that discarded PPEs rising from the pandemic of COVID-19 could end up in our marine ecosystems. The SARS-CoV-2 may be existed on the PPEs surface and enter the water bodies. Improper disposal of PPEs not only results in environmental risk but also causes potential risks to human health due to the consumption of seafood worldwide. In addition, macro-, meso-, and microplastics in aquatic ecosystems can act as possible vectors of pathogens. Also, marine mammals and organisms may be at risk of entanglement and ingestion of latex gloves, which result in intense injuries and death^15^. Plastic waste can make the bulk of mismanaged wastes that interferes with the animal's natural habitat and disturbs those^16^. Biomedical plastic wastes take several years to biodegrade, which allows them to float into terrestrial environments and aquatic ecosystems and threaten human health^17^.

With the COVID-19 outbreak, the generation of waste in Iran has tripled. The volume of hospital waste during the pandemic of COVID-19 in Tehran has reached 100 tons per day, while previously, we had about 60 to 70 tons of hospital waste per day. Therefore, proper disposal of hospital waste in a COVID-19 epidemic is essential. In addition, due to the overburden of hospitals with COVID-19 patients, there is a need to treat infected patients in households. However, there is no management strategy for the proper disposal of generated healthcare waste from households. According to the evidence, medical waste generated in hospitals and healthcare facilities during the COVID-19 pandemic is no different from other infectious medical waste and managed. As a regulation related to medical waste disposal, most wastes are disposed of together in a traditional waste burial. On the other hand, in Iran, often solid wastes are dumped in poorly managed landfills where waste pickers could scavenge for recyclable materials. This can lead to the spreading of the coronavirus. Therefore, to prevent the spread of SARS-CoV-2, an appropriate management strategy is needed to dispose of corona waste.

The COVID-19 pandemic has sparked a rush for plastic with infraction potential. Since most of these plastics cannot be recycled, so has the waste. Growing concern about these wastes like masks, gowns, face shields, safety glasses, protective aprons, sanitizer containers, plastic shoes, and gloves arising from the current COVID-19 pandemic could end up in our aquatic ecosystems. CDC recommended infected plastics have to discard in plastic bags after use and then dumped in trashcans^18^. The unprecedented rises in the number of disposable pandemic wastes lead to provide massive plastic pollution worldwide^19^. This could potentially exacerbate the plastic pollution challenges created by more than 10 million tonnes that have been threatened by our environments^20^. Benson et al.^6^ prepared an international map for specific disposal of mask waste that was considered the most volume of COVID-19 pandemic waste plastics. Doremalen et al. (2020) and Chin et al. (2020) prepared a dataset for COVID-19 pandemic waste, which shows about 266 masks were disposed of per hour^8,21^. This massive amount of pandemic waste leads to an increase in the potential indirect risk of COVID-19 infection^22,23^. In this regard, preparing the special MSW to reduce the COVID-19 pandemic waste is required. The first step in establishing an appropriate MSW is obtaining the generated COVID-19-based pandemic waste volume. This amount of risk-able plastics has to be separated from the main body of municipal waste and disposed of separately. Thus, the specified volume should be transported and isolated and the production volume covers a wide range that must be determined for the past, present, and future. The present study is focused on providing a procedure to determine the COVID-19-based pandemic wastes with high accuracy. As known, the accurate forecast in this field can provide a way to reduce production, safe recovery, and proper burial of pandemic wastes.

The presented article tries to assess pandemic wastes in Iranian metropolises, which play an important role in the indirect spread of COVID-19. In this regard, after providing a comprehensive framework for the COVID-19 spread in megacities in Iran, the predictive model based on a deep neural network was conducted.

## Methods

Deep learning is a subset of machine learning based on artificial neural networks with a representation of learning that attempts to model high-level abstract concepts in the data process using a multiple linear/nonlinear processing layers’ deep graph. Deep learning has developed as deep neural networks (DNN), deep belief networks (DBN), recurrent neural networks (RNN), convolutional neural networks (CNN), restricted Boltzmann machines (RBM), and autoencoders sparse coding (ASC) architectures^24^ which have the exceptional ability to learn the various patterns in data analyses. The DNN (known as dense structural learning) is an artificial neural network (ANN) with multiple layers between the input–output layers which finds the correct mathematical manipulation to turn the input into the output, whether it can be defined as linear or non-linear relationships^25^. The DNN network moves through the layers calculating the probability of each output. The user can review the results and select which probabilities the DNN network should display and return the proposed label. Each mathematical manipulation is considered as a layer, and a complex DNN has many layers, hence the name ‘deep’ networks. Deep architectures include many variants of a few basic approaches where each stage has found success in specific domains^24^.

DNN is a deep learning approach that is used for high accurate classification or prediction based on extracted features from input data (basic or primary dataset). Due to the DNN network capability, by increasing neural layers, the accuracy of the analysis can be increased, so increasing the learning depth. The input data provide the 1st layer of DNN evaluation as a data matrix in which each element has a specific feature value. Hence, the input layer is organized by each DNN layer and unit. These units extract different features from the input data. The output layer was considered as classified/predicted layers from the input data. The middle layers were calculation layers of DNN. Combining these layers in the sequence can extract the desired features and, thereby, classify the input data into the desired classes.

### Study location

Iran is one of the most sensitive countries during the COVID-19 outbreak. The COVID-19 outbreak affected most parts of Iran very fast^26,27^. The growing COVID-19 infection in Iran leads to unpredictable development in the production of pollution-prone waste. On the other hand, the lack of proper locations for landfilling caused this pollution to have a social and trans-social aspect. In this regard, the expansion of COVID-19 and the increase of infected patients caused to increase in the COVID-19-based pandemic wastes rapidly. To determine the impacts of COVID-19 outbreaks on the environment and solve problems in the waste management sector during this pandemic, we need appropriate information about the situation of solid waste in Iran's metropolises. Since only official statistics on infected cases, recovered cases, and mortality are declared by the Ministry of Health and Medical Education of Iran (MHME), it is necessary to contact the municipalities to assess the current state of solid waste management in metropolitan areas. Therefore, extensive field studies were conducted to find the relationship between the number of infected cases and the amount of plastic waste generated in metropolitan areas in both household and hospital wards, and the corresponding graphs were prepared.

### Data resources and preparation

In order to implement the proposed DNN-based model, the basic or primary dataset must first be provided. This dataset will be used to train and tested by the DNN techniques and lead to reaching the prediction goal. The dataset was prepared from 8 Iran metropolises concluded Tehran, Mashhad, Esfahan, Karaj, Shiraz, Tabriz, Qum, and Ahvaz. Data on infected cases is gathered per day in the mentioned metropolises from the beginning of February 27, 2020, to October 10, 2021, based on updates from the website “Worldometer.” These data mostly reflected the COVID-19 spread in the cities. Doing the field survey from both household and hospital plastic waste in each city helped to modify the primary dataset. During the field survey of these megacities, basic information was gathered from hospitals, healthcare centers, and cities’ landfills regarding the volume and type of SUPs and PPEs. Table 2 provides information about the data recourses that were used to enrich the dataset. The provided database categorized the infection cases, PPEs, SUPs, and Test Kits and used medical package volumes in time duration to investigate the pandemic plastic pollution in Iran. All data is classified in rows and columns for each city separately.

**Table 2:** The information about dataset preparation regarding the field survey.

City	Parameter	Unit	Value (average)	Data source
Tehran	COVID-19 infection	Case/day	4464.037	Worldometer
	PPEs	kg/day	14,097.65	MWMU*
	SUPs	kg/day	11,621.03	MWMU
	Test kits	kg/day	30,163.89	IMHME**
	Medical packages	kg/day	45,558.81	IMHME
Mashhad	COVID-19 infection	Case/day	2667.176	Worldometer
	PPEs	kg/day	13,272.11	MWMU
	SUPs	kg/day	10,577.77	MWMU
	Test kits	kg/day	31,914.75	IMHME
	Medical packages	kg/day	40,977.53	IMHME
Esfahan	COVID-19 infection	Case/day	1842.235	Worldometer
	PPEs	kg/day	12,682.44	MWMU
	SUPs	kg/day	9189.781	MWMU
	Test kits	kg/day	23,115.06	IMHME
	Medical packages	kg/day	47,536.48	IMHME
Karaj	COVID-19 infection	Case/day	1743.118	Worldometer
	PPEs	kg/day	12,292.35	MWMU
	SUPs	kg/day	8092.088	MWMU
	Test kits	kg/day	31,497.45	IMHME
	Medical packages	kg/day	35,951.73	IMHME
Shiraz	COVID-19 infection	Case/day	1102.765	Worldometer
	PPEs	kg/day	10,042.53	MWMU
	SUPs	kg/day	8627.327	MWMU
	Test kits	kg/day	27,787.06	IMHME
	Medical packages	kg/day	38,891.01	IMHME
Tabriz	COVID-19 infection	Case/day	811.1765	Worldometer
	PPEs	kg/day	9099.062	MWMU
	SUPs	kg/day	7665.711	MWMU
	Test kits	kg/day	31,560.95	IMHME
	Medical packages	kg/day	32,894.51	IMHME
Qum	COVID-19 infection	Case/day	643.7647	Worldometer
	PPEs	kg/day	8527.540	MWMU
	SUPs	kg/day	6622.454	MWMU
	Test kits	kg/day	26,761.95	IMHME
	Medical packages	kg/day	34,926.61	IMHME
Ahvaz	COVID-19 infection	Case/day	467.0270	Worldometer
	PPEs	kg/day	6640.596	MWMU
	SUPs	kg/day	6105.355	MWMU
	Test kits	kg/day	26,154.13	IMHME
	Medical packages	kg/day	32,377.42	IMHME

*MWMU: Municipality Waste Management Unit.

**IMHME: Iranian Ministry of Health and Medical Education.

After providing the main dataset, this dataset was divided into training and testing sets (80% and 20% of the information, respectively). The training set was used to learn the DNN model, and the test set was used for testing the performance and accuracy of the proposed model.

**Figure 1:**
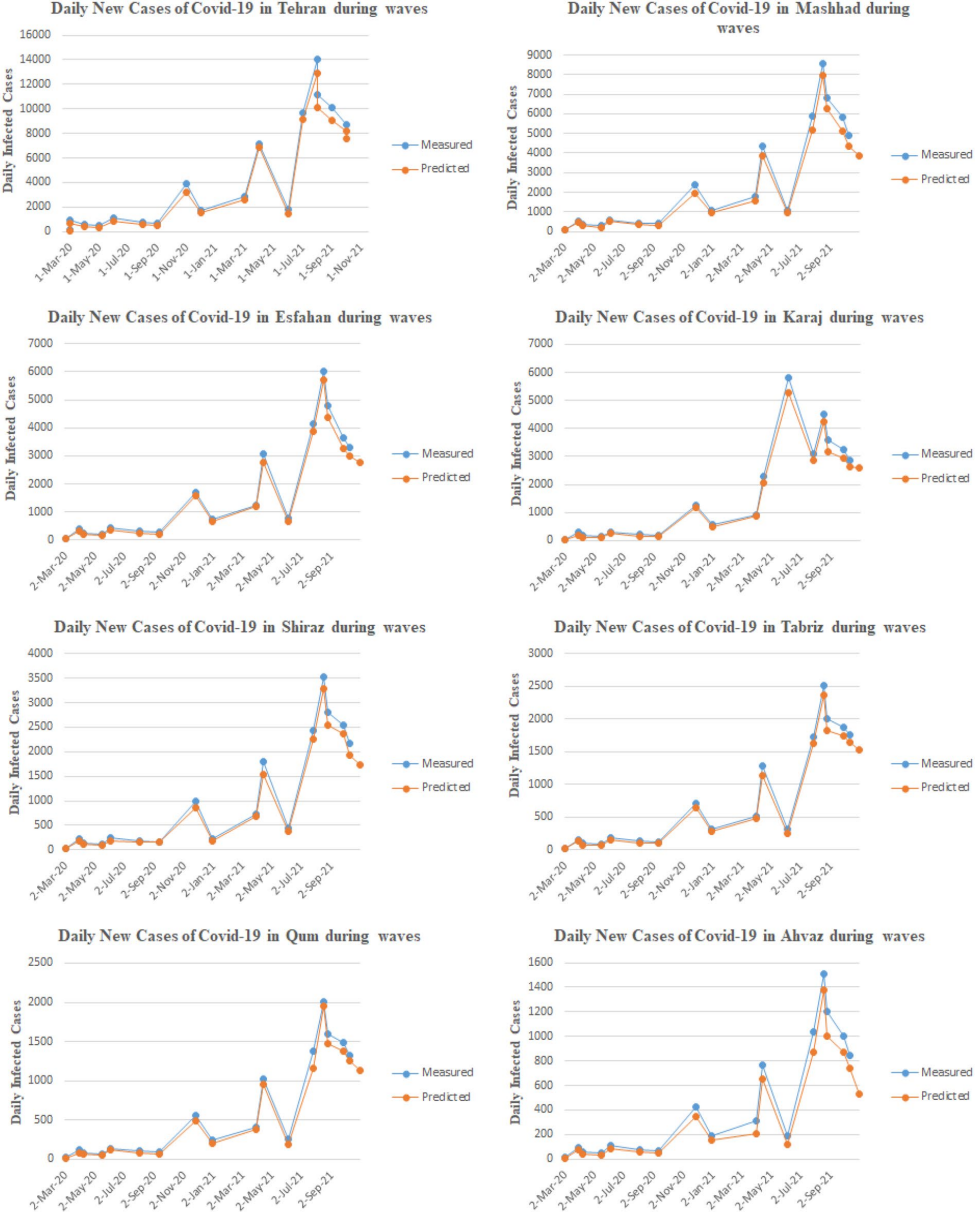
The number of new daily infected cases of COVID-19 in Iran's metropolises.

The number of newly infected cases in Iran's metropolises from the beginning of the first wave that appeared on February 27, 2020, to October 10, 2021, is plotted using Microsoft Excel (Fig. 1). As shown in Fig. 1, at the beginning of the Coronavirus outbreak, the number of infected cases was low in all eight of Iran's metropolises, but because the virus has a rapid spread and due to lack of awareness of how the virus behaves, it has spread rapidly throughout all cities. It led to the beginning of the first wave of COVID-19 in Iran, which reached its wave on March 30, 2020. After passing the first wave, due to the preventive measures of the government and the people becoming more aware and observing the health protocols by them, we witnessed a decrease in the number of infected cases in Iran. Although, due to the reopening of businesses and low observance of health protocols by the people, it did not take long for us to see the start of the second wave again on May 16, 2020, and the number of infected cases increased and reached its wave on June 4, 2020. The third wave of COVID-19 was related to the onset of autumn and the cooling of the weather. In this wave, the number of infected cases increased exponentially, and more patients needed hospitalization and intensive care. Coinciding with the emergence of the new coronavirus mutation, known as the British Variant, the fourth wave of COVID-19 began and remained in Iran until June 4, 2021. Unfortunately, due to the spread of the Delta Variant, Iran is currently in the fifth wave of COVID-19. The number of infected cases in this wave reached a record 50,228 cases on October 10, 2021.

### Field survey and ground investigations

Iran is one of the countries with a high prevalence of COVID-19. As of October 10, 2021, there had been 5,754,047 confirmed cases and 123,498 deaths in Iran, which made this country come 8th ranked in the world (Worldometer website). Along with other issues such as non-compliance with health protocols, not implementing social distancing, and unsafe traveling during the COVID-19 pandemic, improper handling of corona waste in developing countries, including Iran, increases the possibility of Coronavirus propagation. In Iran, with more than 85 million people, over 18 million tonnes of municipal solid waste (MSW) annually are generated. Only 8% of MSW are recycled by legal framework due to poor separation programs implemented all over the country. Therefore, hazardous household waste, including medical waste, is mixed with general household waste and can have health and environmental problems^28^. In this regard, the presented study provides an extensive field survey from the main hospitals and municipal waste management units in megacities to provide the relevant information used in the primary dataset. This information, after pre-processing, is used in the prediction process. During the pre-processing stage, the non-relevant data, like the waste volume of not pandemic like non-organic wastes, food waste, metals, etc., was removed from the database. The main focus was on pandemic plastics like PPEs, SUPs, and medical wastes that are potentially prone to pollution.

### DNN model implementation

After providing the primary dataset that was used as a basic database of the COVID-19 spread and pandemic plastic usage in various megacities in Iran, the dataset was randomly divided into testing and training sets. In the next stage, the model was trained and tested regarding the learning rate. Considering the test/train ratio is important for the model learning rate, that is, the response to the estimated error each time the model weights are updated. In fact, the learning rate controls how quickly the model is adapted to the problem. Lower learning rates require more training epochs as smaller changes are made to the weights at each update, whereas larger learning rates result in rapid changes and require fewer training epochs. Specifically, the learning rate is a configurable hyperparameter used in the training of neural networks that has a small positive value, often in the range between 0.0 and 1.0. The learning rate used in this study was selected by optimizers, which for 0.01 and no momentum were scheduled via callbacks in Keras support. To this end, the DNN model was run for 700 iterations (epochs) using the training and validation datasets.

**Figure 2:**
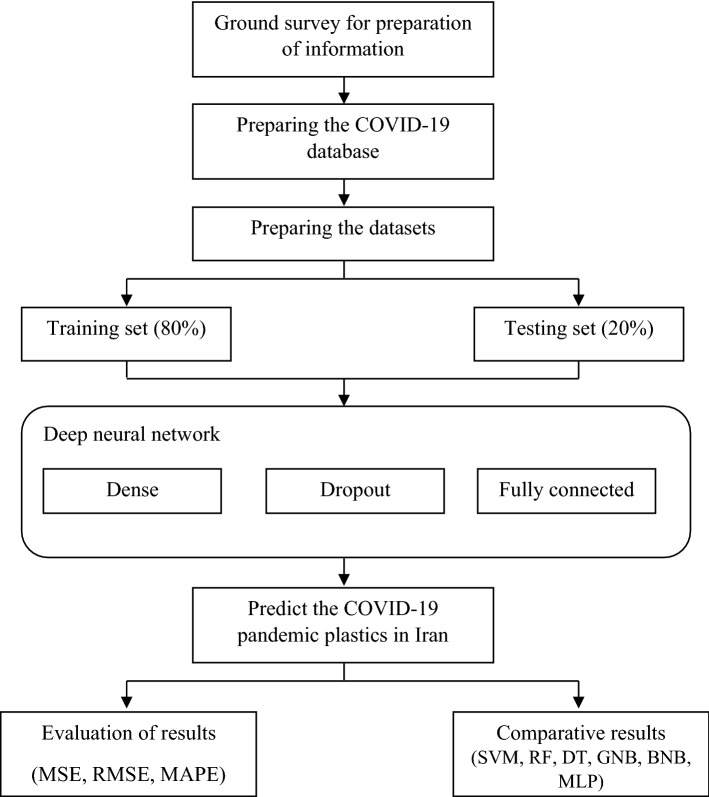
The processing flowchart of the DNN predictive model.

This database randomly divided into the testing and training data sets which are cover 20% and 80% of the primary database, respectively. Figure 2 is illustrated the processing flowchart of the DNN model implementation. The DNN-based predictive model is used to forecast the riskable pandemic plastic pollution for future events.

### Performance evaluations

The performance of the proposed methodology was estimated based on both the confusion matrix and statistical error estimators such as mean squared error (MSE), root means square error (RMSE), and mean absolute percentage error (MAPE). The performance matrix is a specific table that visualizes the performance of a prediction algorithm based on its predicted values, and it contains the sensitivity, specificity, and 1-specificity parameters. For classification tasks, the terms true positives (TP), true negatives (TN), false positives (FP), and false negatives (FN) compare the results of the classifier in question with trusted external judgments^25^. Precision (TP/[TP + FP]), also called the positive predictive value, is the fraction of relevant instances (TP) among the retrieved instances. Also, recall (TP/[TP + FN]) is the total fraction of relevant instances.

Both precision and recall, therefore, are based on measures of relevance^29^. Accuracy can be a misleading metric for imbalanced datasets. For example, for a prediction set with 90 positive and 10 negative values, classifying all values as negative gives a 0.90 (90%) accuracy score. The f1-score which known as the harmonic factor (F1 = 2 × [precision × recall]/[precision + recall]) provides approximately the average of the precision and recall values when they are close and is more generally the harmonic mean.

The overall accuracy represents the probability that an individual will be correctly classified by a test; that is, the sum of TP plus TN divided by the total number of the individuals tested.The application of the performance matrix helps to characterize the trustworthiness of the classifiers in question^24^.

To estimate the error estimators from the confusion matrix, the mean squared error (MSE), root mean square error (RMSE) and mean absolute percentage error (MAPE) was used to measure the model accuracy. In statistics, MSE, RMSE, and MAPE are considered as an estimator to measure the average of the squares of the errors between the estimated values and the actual value. In machine learning, these errors represent the empirical risk of the average loss on an observed dataset which indicates the rate of predictive model accuracy.

### Verifications

The common intelligence learning-based classifiers are used for justification of applied DNN model to verification of modeling. In this regard, the k-nearest neighbors (k-NN), decision tree (DT), random forests (RF), support vector machines (SVM), Gaussian naïve Bayes (GNB), logistic regression (LR), and multilayer perceptron (MLP) methods were selected to comparative subjects for prepare confusion matrix. In the machine learning field and specifically in a statistical classification problem, a confusion matrix is used to investigate the performance of applied algorithms especially supervised learning. The confusion table indicates the degree of visualization based on information retrieval documents which allows more detailed analysis than a mere proportion of correct classifications (accuracy). In the matrix’s context, precision (represents the positive and negative predictive values in numeral diagnostic tests), recall (represents the performance of binary classifications sensitivity), and f1-score (harmonic factor) are defined as relevant documents. The comparative algorithms were used as justification^30^. The above classifiers were used for verification of DNN based method by providing the comparative confusion tables. Also, the receiver operating characteristic (ROC) is used to control of mentioned predictive models’ performances. The ROC curve is a graphical description that shows the diagnostic ability of a binary classifier system as its discrimination threshold is varied. As a result, the overall accuracy and area under the curve (AUC) from the confusion matrix and ROC curve represent the accuracy of the classifiers. All models from DNN to verification classifiers are tested by both the confusion matrix and ROC to obtain the performance status of the methods.

## Results

**Figure 3:**
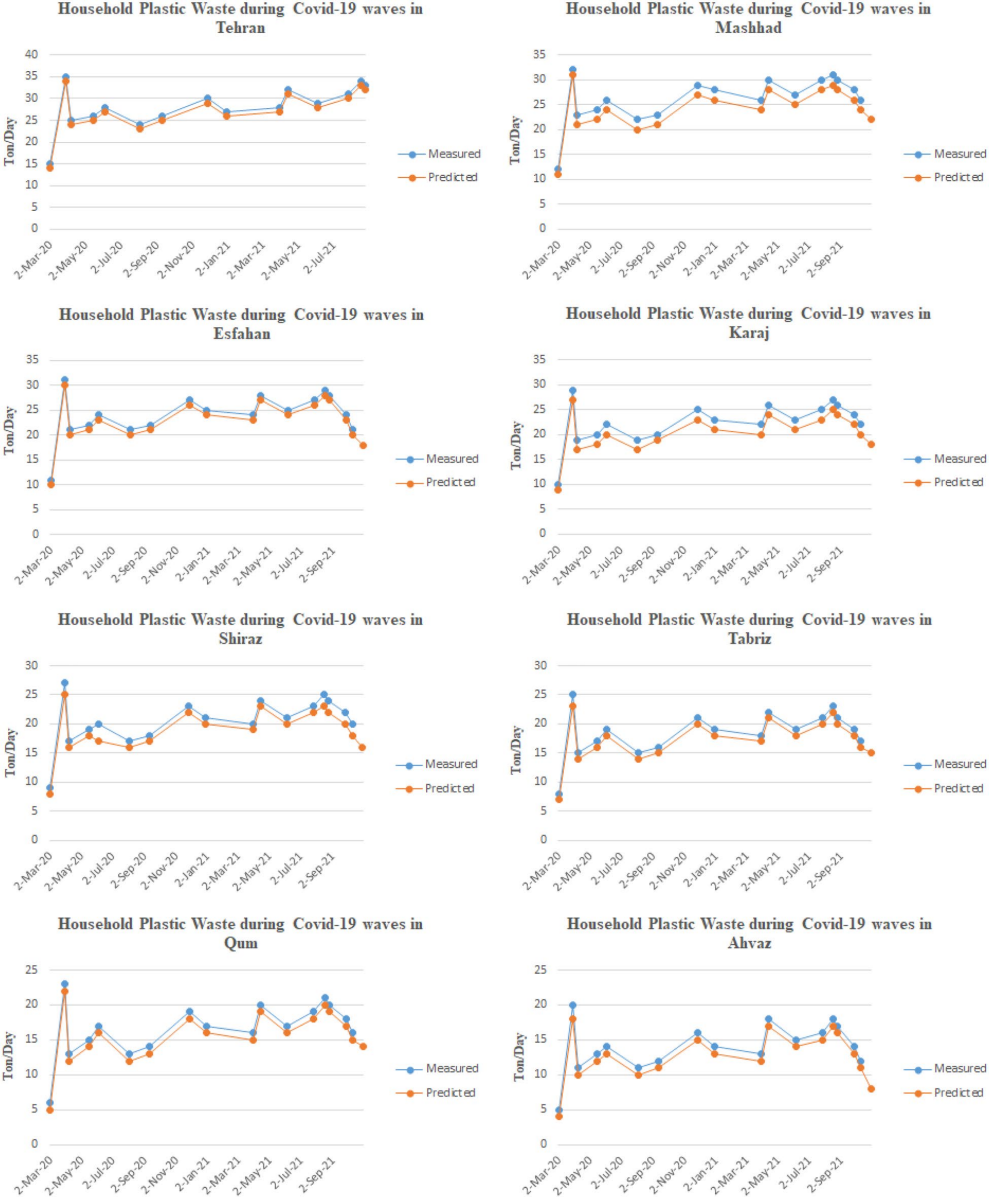
The amount of household plastic waste caused by COVID-19 in Iran's metropolises.

**Figure 4:**
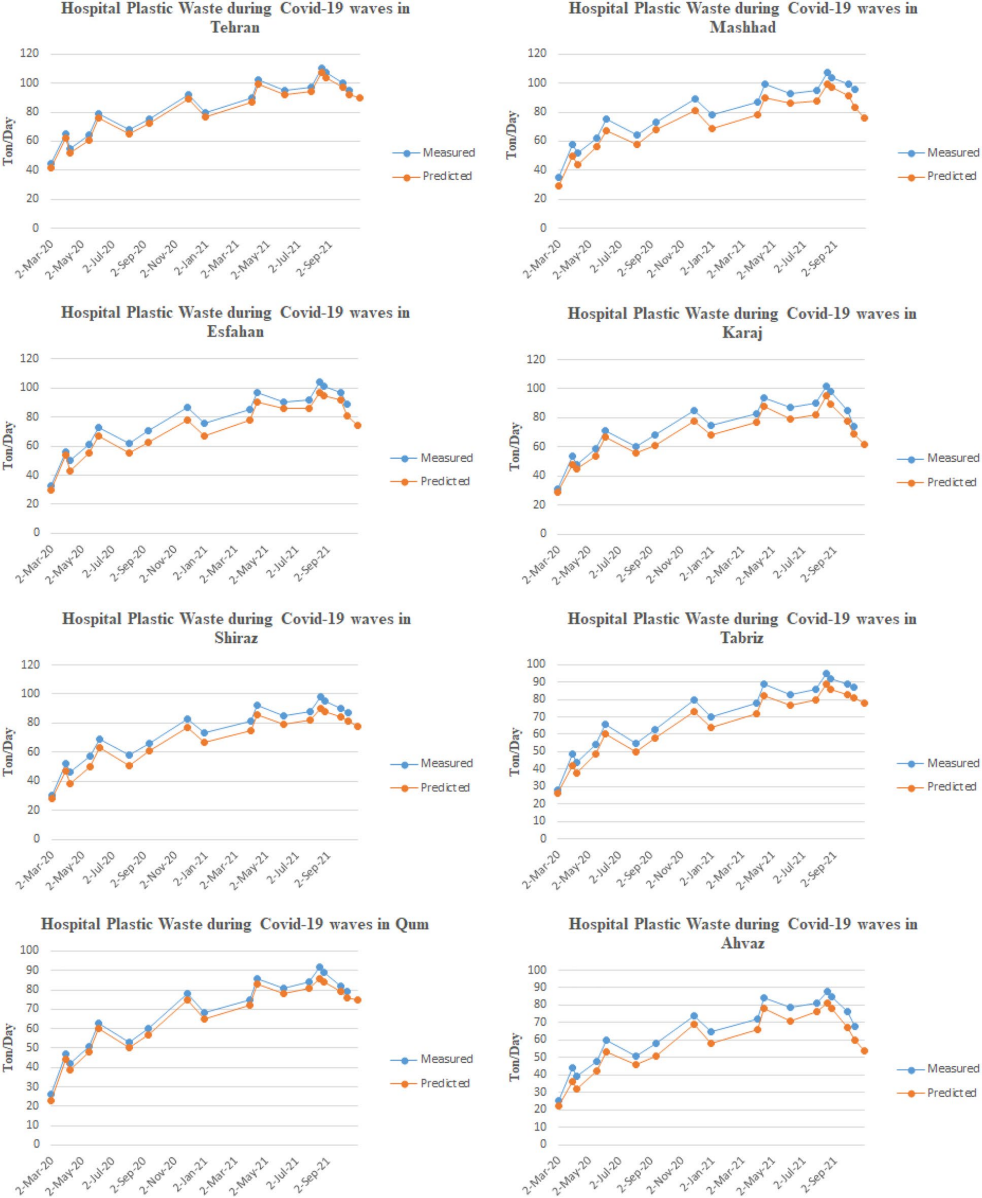
The amount of hospital plastic waste caused by COVID-19 in Iran's metropolises.

The number of newly confirmed cases and the amount of household and hospital plastic waste caused by COVID-19 in all 8 metropolises of Iran have been obtained, and the results are shown in Figs. 3 and 4. As shown in Fig. 3, at the beginning of the outbreak of COVID-19 in Iran in all eight metropolises, the generation of household plastic waste increased unprecedentedly. The reason was fear of the virus and excessive use of disposable plastic items such as disposable gloves, plastic bags, masks, and face shields. Also, with lockdown cities at the beginning of the outbreak, more household waste was generated. By subsiding the initial wave of the disease, people's sensitivity decreased, but due to using the PPEs, the generation of household plastic waste has increased compared to previous years. On the other hand, as shown in the graphs, during the peak of the disease in the first to fifth waves, the production of plastic waste increases significantly, which is related to the stress caused by COVID-19, and people usually use more PPEs during the waves of COVID-19. Figure 4 shows the generation of hospital plastic waste. As shown in the graphs, at the beginning of the disease outbreak in Iran, the production of hospital waste compared to household waste did not increase significantly. However, with the progression of the disease and more people referring to medical facilities and hospitals, the process of waste generation has increased, and at the same time, with the peak of the disease in multiple waves, the production of hospital waste has increased. This increase is related to the consumption of PPEs by medical staff such as doctors, nurses, etc., as well as the plastic equipment used by COVID-19 patients.

**Figure 5:**
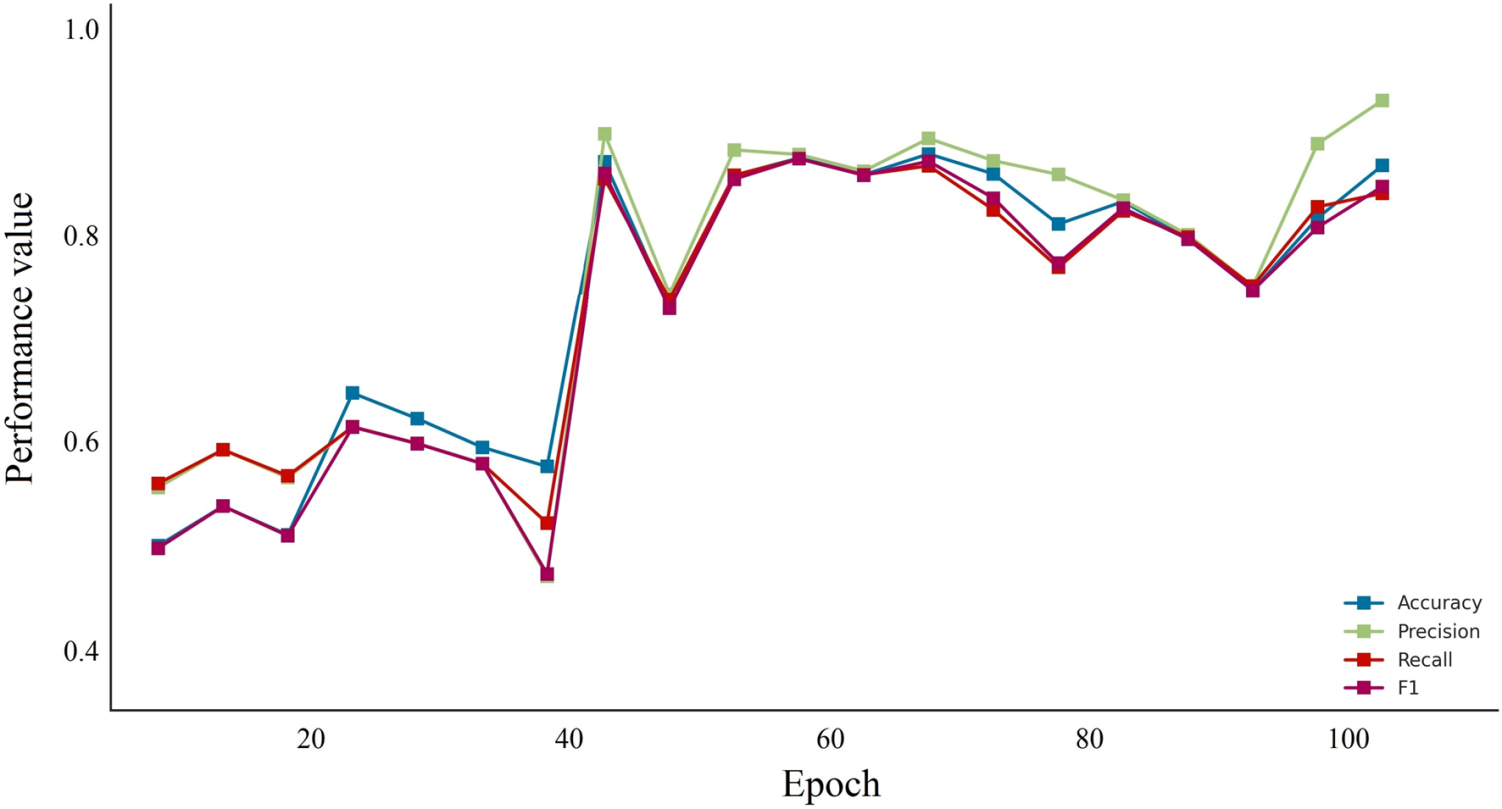
The DNN model performance evolution for COVID-19 pandemic plastic in the first 100 epoch.

**Figure 6:**
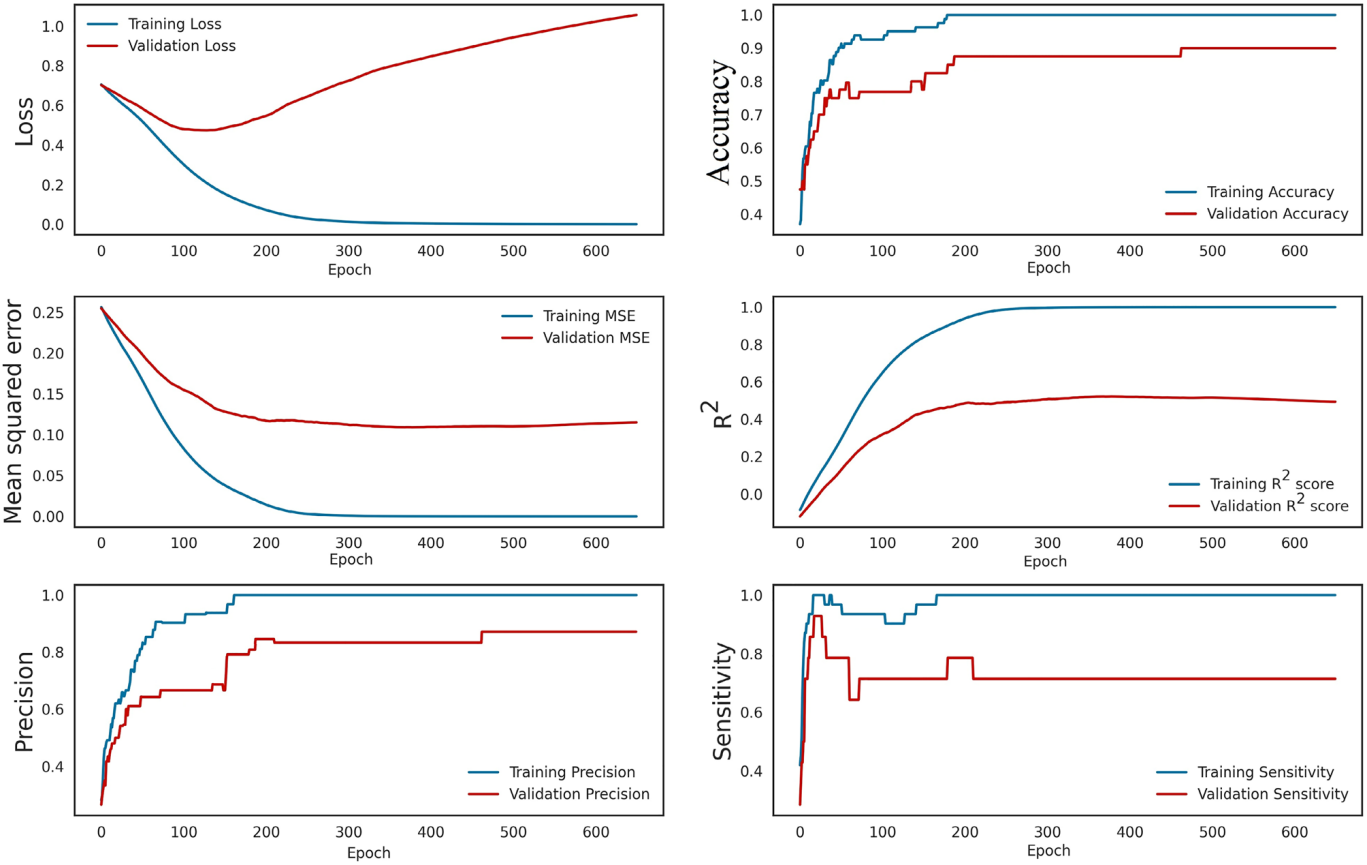
The error rate and confusion matrix for DNN predictive model.

According to these figures, it can be seen that Iran has faced different waves of COVID-19 epidemics, which has led to an exponential increase in the use PPEs. There is a logical relationship between increasing PPEs and increasing COVID-19 contagion waves in Iran. Thus, by using a prediction method to forecast the increase/decrease level of pandemic plastic wastes for future events. Nevertheless, the information about COVID-19 contagion waves from February 27, 2020, to October 10, 2021, and the PPEs usage during this duration were utilized as the primary database for the DNN-based predictive model. By applying the predictive model to measured data, it can be mentioned the model provides a suitable prediction of PPEs expansion in different cities. Figure 5 is illustrated the results of the DNN predictive model performance value for the first 100 stages of evaluation. According to this figure, the model performance evaluation is conducted properly, and the evaluation criteria reached 0.9 in the first stages. Figure 6 is provided the predictive model results of the confusion matrix during the forecasting of the COVID-19 pandemic wastes expansion in Iran's metropolises. According to these figures, it can be mentioned the DNN-based model provides high-accurate results to predict plastic pollution.

**Figure 7:**
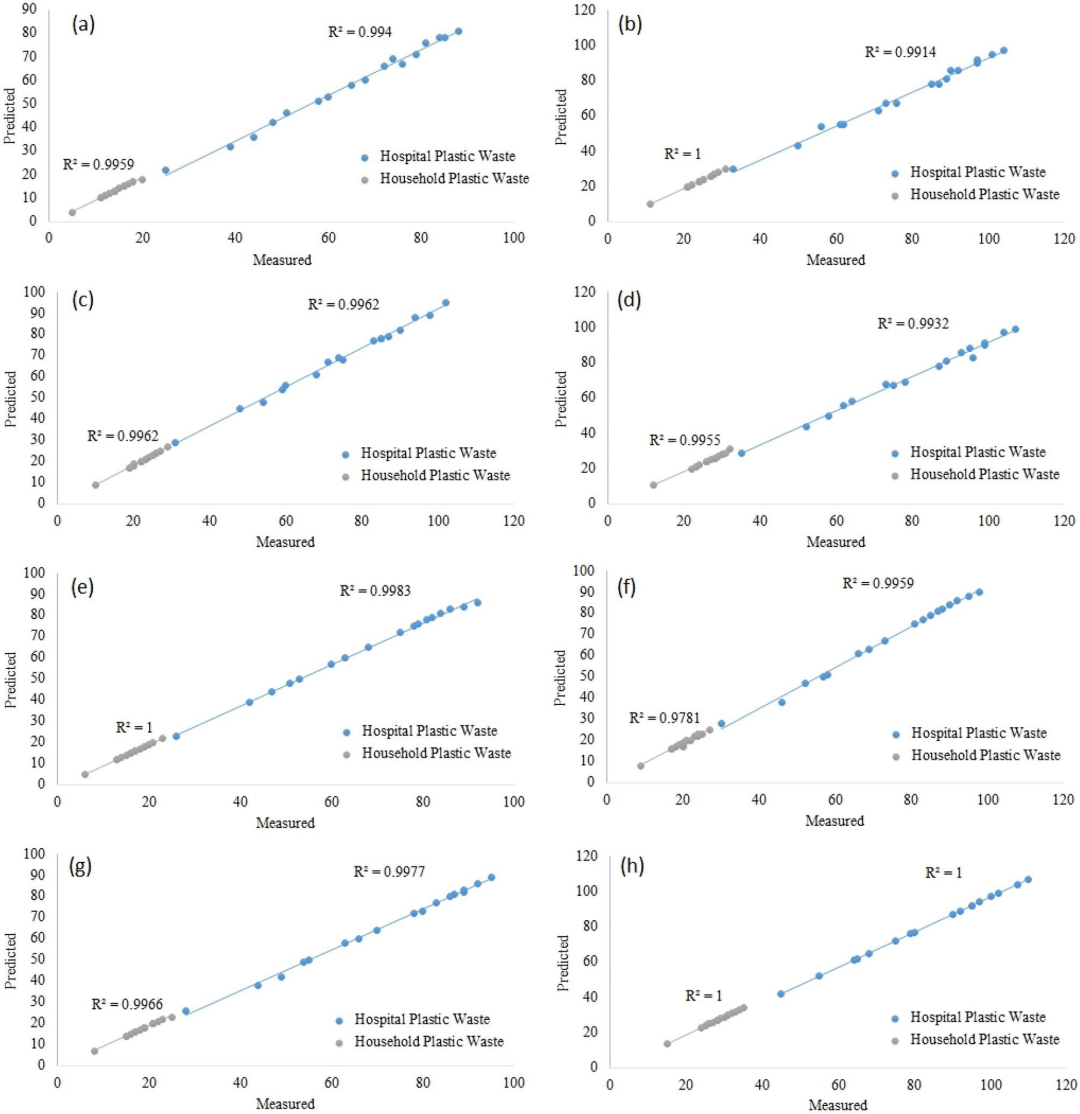
The regression metrics for DNN predictive model: (**a**) Ahvaz, (**b**) Esfahan, (**c**) Karaj, (**d**) Mashhad, (**e**) Qum, (**f**) Shiraz, (**g**) Tabriz, (**h**) Tehran.

Figure 7 illustrates the prediction regression variations that were conducted on the database by the DNN model for megacities of Iran. The presented figure provided a linear regression analysis for measured and predictive values of the used PPEs for different studied cities and estimated the R-square values in hospital and household plastic wastes. As seen in this figure, the regression between measured and predicted values indicates the DNN model’s high capability. As a justification of the DNN model used, the common classifier concluded as k-NN, DT, RF, SVM, GNB, LR, and MLP methods. The justification procedure utilized to prepare the comparative confusion matrix was used for a measure of methods performance. The confusion matrix and ROC curve are used as verification. Precision (also called positive predictive value) is the fraction of relevant instances among the retrieved instances, while recall (also known as sensitivity) is the fraction of relevant instances that were retrieved. Both precision and recall are therefore based on relevance. So, the precision represents the total of true prediction, and recall has presented a sensitivity of the analysis. Figure 6 provides information about the precision, sensitivity, and accuracy of the DNN model during the training and validation process. Also, in order to evaluate the model capability, the error rates were evaluated for all predictive models shown in Table 3. The error rate indicated the predictive model's accuracy. The MSE, RMSE, and MAPE values are obtained for the various classifiers. According to this table, the DNN model outperformed the benchmark methods.

**Table 3:** Estimated error rates for different predictive models.

Classifier	MSE	RMSE	MAPE
k-NN	0.42718541	0.41025881	0.50487288
Decision tree	0.45047509	0.45527133	0.67085132
Random forests	0.29886415	0.30175298	0.31253664
SVM	0.28157449	0.27168503	0.25416574
GNB	0.33689459	0.38012520	0.35336942
MLP	0.10746354	0.13714221	0.17456325
Logistic regression	0.25776357	0.27596413	0.25976830
DNN	0.02435120	0.02798154	0.02595136

## Discussion

The deep neural network (DNN) is a type of machine learning model that is capable of learning complex patterns in data and can be applied to a wide range of tasks. They have been used in many fields, including image and speech recognition, natural language processing, and forecasting, to name a few. The presented study attempted to investigate DNN to predict COVID-19 pandemic plastic waste expansion in eight megacities in Iran, which is considered the main objective of the study. The algorithm evaluated PPEs and SUPs generation regarding the COVID-19 pandemic spread in Iran during that specific time. The prediction was implemented on the primary inventory database, which was provided based on digital and field (site) surveys from the target cities; this was divided randomly into training (80%) and testing (20%) sets for the analysis. The DNN-based predictive model was compared to the benchmark models regarding performance and accuracy which results show a significant capability of the algorithm to predict accurately. The high accuracy achieved by the proposed DNN predictive model over other comparative classifiers as well as fewer errors rate. As a notification, this study has some limitations that could be considered in future research. These limitations can be addressed as follows:

- The primary database was provided based on released information from authorities and large hospitals/ medical centers, and urban services of the municipality from the megacities, so the input data is limited.
- The predictive model requires many strong processors to analyze the inputs, so the processing stages are time-consuming and slow.

The predictions based on the time series have shown good agreement with the measured data, as shown in Figs. 1, 3, and 4. The forecasting results have indicated that the using and producing pandemic wastes are compatible with the spread of COVID-19 across entire cities. Household plastic waste, in particular, show a decreasing trend. However, hospital waste is more closely linked to the overall trend of infections.

Referring to the justification process, the proposed model reached the highest accuracy (0.96) with precision (0.93) rather than other classifiers concluded k-NN (0.70 accuracy and 0.67 precision), DT (0.85 accuracy and 0.75 precision), RF (0.82 accuracy and 0.73 precision), SVM (0.82 accuracy and 0.60 precision), GNB (0.82 accuracy and 0.81 precision), MLP (0.77 accuracy and 0.84 precision), and LR (0.74 accuracy and 0.66 precision). Also, the obtained error rate indicated the DNN model was operated with less error than others which is MSE = 0.024, RMSE = 0.027, and MAPE = 0.025. The other classifiers error rates are k-NN (MSE = 0.427, RMSE = 0.410, MAPE = 0.504), DT (MSE = 0.450, RMSE = 0.455, MAPE = 0.670), RF (MSE = 0.298, RMSE = 0.301, MAPE = 0.312), SVM (MSE = 0.281, RMSE = 0.271, MAPE = 0.254), GNB (MSE = 0.336, RMSE = 0.380, MAPE = 0.353), MLP (MSE = 0.107, RMSE = 0.137, MAPE = 0.174), and LR (MSE = 0.257, RMSE = 0.275, MAPE = 0.259). Regarding the benchmark classifiers used in the study for verification, the models have shown the superiority of the proposed DNN procedure. Finally, the ROC curves were used to evaluate the overall accuracy and degree of the capability for each machine learning-based predictive model. According to the results, the proposed method reached the highest overall accuracy (AUC = 0.969). Other classifiers reached k-NN (AUC = 0.809), DT (AUC = 0.828), RF (AUC = 0.828), GNB (AUC = 0.908), MLP (0.779), and LR (AUC = 0.742).

## Conclusions

The global outbreak of COVID-19 is providing a high alert situation worldwide and lead to an increase in using personal protective equipment (PPE) based on WHO and CDC recommendations. These PPEs caused the extension of medical waste (named pandemic plastic pollution) with high risk-able as COVID-19 infection potential in the environment, which is required special MSW management. Addressing the COVID-19-based pandemic waste generation in the world and Iran provide a huge concern about indirect COVID-19 spread. Providing information about the pandemic plastic pollution volumes is the first step in MSW management for such risk-able wastes. The presented study attempted to provide a proposed predictive model based on deep learning and deep neural network (DNN) framework. The aim of the predictive model is to prepare an accurate view of COVID-19-based pandemic plastic expansion in the megacities of Iran. In this regard, the pandemic spread and PPEs usage data were gathered from February 27, 2020, to October 10, 2021, and used as the primary database for the predictive model. The database used for DNN-based modeling regarding the predicting of MSW volume for pandemic plastic expansions. The results of the research can be categorized as follow:

1. The pandemic plastics (e.g., masks, gloves, aprons, and bottles of sanitizers) are consequences of COVID-19 pandemic-infected waste, which is significantly increased at the global level. These hazardous wastes play an important role in environmental pollution and indirectly spread COVID-19. The pandemic plastics expansion leads to global concern about COVID-19-based risk-able wastes, and providing special MSW management is necessitating urgent prevention to control the pandemic spread.
2. The prediction of the pandemic plastics’ volume can be used for appropriate MSW management, considered the first step to controlling the indirect pandemic spread. In this regard, the application of artificial intelligence techniques received huge attention due to the highly accurate results. In the meantime, the application of deep learning approaches provides more efficient predictive models.
3. The deep learning procedure (DNN) was used for the prediction of COVID-19-based pandemic plastics expansion in megacities of Iran. The model was implemented on a comprehensive database for forecasting the waste variations with respect to COVID-19 epidemic waves in Iran. The predictive model is controlled by a confusion matrix to evaluate the model's performance. According to the confusion matrix, the DNN model provides 96% accuracy and 93% precision. Also, the estimated error rate is MSE = 0.024, RMSE = 0.027, and MAPE = 0.025, which indicates the high performance of the predictive model regarding other machine learning-based algorithms.
4. The common classifiers used for justification for DNN predictive models such as k-NN, DT, RF, SVM, GNB, MLP, and LR algorithms were selected for comparative subjects. According to the performance evaluations, the models operated under k-NN (0.70 accuracies and 0.67 precision), DT (0.85 accuracies and 0.75 precision), RF (0.82 accuracies and 0.73 precision), SVM (0.82 accuracies and 0.60 precision), GNB (0.82 accuracies and 0.81 precision), MLP (0.77 accuracies and 0.84 precision), and LR (0.74 accuracies and 0.66 precision). Based on errors table obtained for these classifiers concluded NN (MSE = 0.427, RMSE = 0.410, MAPE = 0.504), DT (MSE = 0.450, RMSE = 0.455, MAPE = 0.670), RF (MSE = 0.298, RMSE = 0.301, MAPE = 0.312), SVM (MSE = 0.281, RMSE = 0.271, MAPE = 0.254), GNB (MSE = 0.336, RMSE = 0.380, MAPE = 0.353), MLP (MSE = 0.107, RMSE = 0.137, MAPE = 0.174), and LR (MSE = 0.257, RMSE = 0.275, MAPE = 0.259).
5. According to the confusion matrix and statistical error estimators’ results, the DNN model has achieved more accuracy than justification methods which are indicated the capability and high performance of the DNN predictive model over other methods.
6. The receiver operating characteristic (ROC) curve analysis results of all models to evaluate the degree of the capability that indicates the DNN model (AUC = 0.969) was the highest score than others which contains k-NN (AUC = 0.809), DT (AUC = 0.828), RF (AUC = 0.828), GNB (AUC = 0.908), MLP (0.779), and LR (AUC = 0.742).

## Data Availability

All data generated or analyzed during this study are included in this published article.erest.
